# Surgical management of abdominal compartment syndrome; indications and techniques

**DOI:** 10.1186/1757-7241-17-17

**Published:** 2009-04-14

**Authors:** Ari Leppäniemi

**Affiliations:** 1 Department of Surgery, Helsinki University Hospital, Haartmaninkatu 4, PO Box 340, 00029 HUS, Helsinki, Finland

## Abstract

The indications for surgical decompression of abdominal compartment syndrome (ACS) are not clearly defined, but undoubtedly some patients benefit from it. In patients without recent abdominal incisions, it can be achieved with full-thickness laparostomy (either midline, or transverse subcostal) or through a subcutaneous linea alba fasciotomy. In spite of the improvement in physiological variables and significant decrease in IAP, however, the effects of surgical decompression on organ function and outcome are less clear. Because of the significant morbidity associated with surgical decompression and the management of the ensuing open abdomen, more research is needed to better define the appropriate indications and techniques for surgical intervention.

## Introduction

Sustained increase in intra-abdominal pressure (IAP) defined as intra-abdominal hypertension (IAH) can with ensuing onset of organ dysfunction lead to abdominal compartment syndrome (ACS) [1,2]. If not timely treated, it can lead to serious organ failures and even death. Whether the cause is in the abdomen (primary ACS) or elsewhere (secondary ACS), prevention is of utmost importance. Fluid resuscitation is a very important cause of IAH when high volumes are necessary, such as in patients with severe acute pancreatitis (SAP). In these patients, the excessive use of crystalloids should be avoided.

The first line of treatment is always nonoperative [3]. Nasogastric decompression is useful in patients with gastric dilatation or ileus. Short term use of neuromuscular blocking agents has been used in appropriate circumstances. Removal of excessive fluids with diuretics can be tried, but extracorporeal techniques, such as continuous hemodiafiltration, are more effective in rapidly removing excess fluid. Finally, percutaneous drainage of intraperitoneal fluid collections is a simple and more effective way to reduce intra-abdominal volume.

If nonoperative measures fail to relieve ACS, surgical decompression should be considered. Due to the severe morbidity associated with all forms of surgical decompression, the indications, timing and technique used should be carefully evaluated.

## Indications for decompression

There is no uniform consensus on the indications for surgical decompression in ACS. In addition to IAP values, the cause, time-frame and possible need for further laparotomy should be considered. As a general rule, when non-surgical interventions fail to turn around the progressive deterioration of organ dysfunctions in the presence of fulminate ACS, surgical decompression is justified.

## Timing of decompression

Clinical experience seems to indicate that early decompression is more effective and associated with lower mortality than delayed decompression performed after several days after the onset of ACS, but no comparative studies exist. Obviously, it depends on the time when ACS develops. Usually ACS is an early phenomenon and prompt decompression without significant delay is appropriate. However, if ACS is caused by a later event, such as infection of the peripancreatic necrosis in SAP, for example, decompression at that stage is warranted and often combined with necrosectomy. There are no studies on prophylactic surgical decompression, but preventing ACS by leaving the abdomen open in high risk patients, such as patients undergoing damage control surgery for trauma or patients operated for ruptured abdominal aortic aneurysms, is sensible.

## Techniques

**Figure 1:**
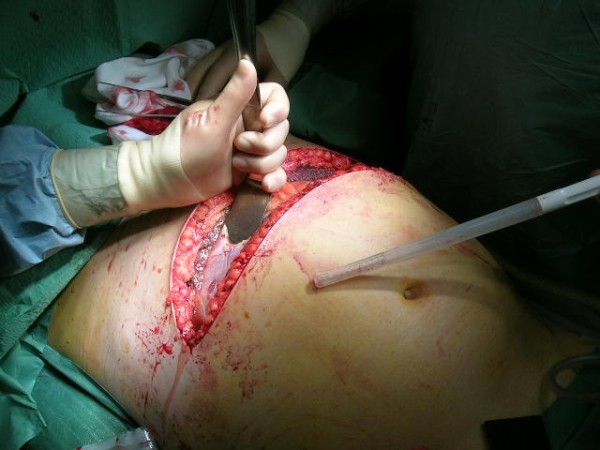
**Decompressive transverse laparostomy**.

When selecting the optimal technique for surgical decompression, previous already existing recent abdominal incisions (wounds) are preferable. If a patient has undergone a midline laparotomy, utilizing the same incision for decompression is obviously the best choice. In patients with secondary ACS and particularly those without previous abdominal incisions, several options exist. The most commonly used method for surgical decompression is the midline laparostomy [4]. All layers (skin, fascia, peritoneum) are divided through a vertical midline incision extending from the xiphoideum to the pubis with a few centimeters of fascia left intact at both ends to facilitate subsequent closure or late reconstruction. Alternatively, a transverse bilaterally extended incision few centimeters below the costal margins can be used to perform a full-thickness laparostomy [5] (Fig. 1). A third method utilizes three short horizontal skin incisions to perform a subcutaneous anterior abdominal fasciotomy at the linea alba (SLAF) with the peritoneum left intact [6].

## IAP decrease after full-thickness laparostomy

All techniques have been shown to reduce IAP. The majority of evidence is based on experience from patients with SAP. In a collective of analysis of 161 patients undergoing surgical decompression via midline laparostomy, the mean reported IAP before surgical decompression was 34.6 mmHg and fell to 15.5 mmHg after decompression (p < 0.001) [4]. The experience with transverse laparostomy is still scarce, but a case report showed the decrease from 23 mmHg to 10 mmHg [5].

## IAP decrease after fasciotomy

The original report of two patients utilizing the SLAF method showed a decrease of IAP from 30 mmHg to 14 mmHg and 35 mmHg to 23 mmHg, respectively [6]. Another report showed a decrease from 27 mmHg to 11 mmHg [7]. In analyzing the first 10 patients with SAP undergoing SLAF at the Meilahti hospital in Helsinki, the mean preoperative IAP was 31 (range 23–45) mmHg and fell to 20 (10–33) mmHg immediately postoperatively with a mean decrease of 10 (2–17) mmHg [Leppäniemi A, unpublished data]. The initial decompressive effect was deemed sufficient in 7 patients, out of whom 2 developed recurrent ACS and underwent completion laparostomy.

## Selection of decompressive method

Because there are no randomized studies comparing different surgical decompression techniques, the selection of the technique has to be individualized and based on common sense and weighing the pros and cons of each technique. The midline laparostomy is relatively safe, easy to perform and nearly always effective. Although early complications, such as intestinal fistulas, have been greatly reduced with careful management and better understanding of the open abdomen, there is a high risk of persistent open abdomen requiring split-thickness skin grafting and delayed reconstruction of the abdominal wall (planned hernia approach). Recently, the use of temporary mesh to facilitate gradual fascial closure has decreased the planned hernia rate.

Transverse laparostomy seems to be effective, is little more time-consuming and could have a higher rate of fascial closure. In addition, same principles of managing the open abdomen can be applied without additional equipment. The major disadvantage is the loss of abdominal and back extensor muscle functions, if fascial closure can not be achieved. This would require complex reconstruction procedures including innervated free flaps that not only restore continuity but also the functional integrity of the abdomen [8].

SLAF is effective in about 50–70%, prevents open abdomen and its related complications, but is always associated with a subsequent hernia. In the acute phase, the cost effectiveness due to lesser need of nursing care and reoperation resources is a significant advantage.

## Where to decompress

From a surgical technical point of view, the best place to perform a decompressive operation is the operation theatre. It provides adequate aseptic conditions, lighting, equipment and personnel, and is ergonomically better for the surgeon. However, in urgent situations, decompression can and sometimes should be performed in the Intensive Care Unit (ICU). Even under less urgent conditions, a surgical team with proper equipment can go the ICU and perform the operation there without additional risk to the patient. The benefit of not requiring the transfer of a critically ill patient with multiple monitors and ongoing medications is obvious, and it also saves operation room time.

## Effects of surgical decompression on organ failure and outcome

In a collective review of 250 patients undergoing midline laparostomy, decompression had an overall positive effect on hemodynamic, respiratory and renal functions [4]. Central venous pressure and pulmonary artery pressure decreased, most likely caused by the direct effect of the decrease in IAP on the thoracic cavity. Cardiac function improved in the majority of the patients. There was an improvement in PaO_2_/FIO_2 _ratio and decrease in peak airway pressure, but the respiratory function remained severely impaired in most patients. Significant improvement in the urinary output was observed in all but two studies.

At our institution, among the 26 patients with SAP undergoing surgical decompression for ACS during the past 6 years, mostly a full-thickness midline laparostomy, there was no significant difference between pre- and postoperative organ dysfunctions score [Leppäniemi, unpublished data]. The PaO_2_/FIO_2 _ratio increased in 50% and decreased in 50% of the patients. Daily urinary output increased by >200 ml in 7 patients (27%), and 3 patients avoided renal replacement therapy. The overall mortality rate was 46% with preoperative renal failure (p = 0.045), lower preoperative IAP (p = 0.039) and late (median 7 days) decompression (p = 0.005) being associated with increased risk of death. It is noteworthy that all 8 patients undergoing surgical decompression more than 3 days post-admission died.

It seems that in most cases ACS is an early phenomenon requiring prompt action. If nonoperative techniques fail to provide significant decrease in the IAP and improvement in organ functions, surgical decompression should be performed without additional delay. As institutional experience in the management of ACS increases, the delay from onset of the problem to interventions tends to decrease.

## Complications of surgical decompression

Although postoperative bleeding and infection can occur after any surgical procedure, they are rare after decompressive laparostomy. Recurrent ACS can develop after too eager closure attempts. However, the major source of morbidity is associated with the management and complications of the open abdomen.

## Management of the open abdomen

In view of the complicated and often fatal outcome of patients ending up with a persistent open abdomen after multiple reoperations, often with entero-atmospheric fistulae and persistent infection, the "hostile abdomen" scenario, extreme caution and care should be administered when managing patients with open abdomen [9].

The ideal cover of the abdominal contents after leaving the abdomen open should protect the viscera, avoid fistulas, be easy to apply and remove, allow easy nursing care, should not damage the fascia or the skin, be readily available and inexpensive, and maintain the abdominal domain. In addition, the preservation of the accessibility to the abdominal cavity and the feasibility of gradual closure of the abdominal wall are important.

The easiest method to cover the abdominal viscera after decompression is a plastic silo (Bogota bag) which is inexpensive, readily available and which preserves the intact fascia when sutured to the skin edges. However, because the plastic silo or some other form of temporary abdominal closure allows the fascial edges to retract laterally, the abdominal cavity looses part of its volume resulting in difficult fascial closure under significant tension especially, if the closure is delayed beyond the first week.

**Figure 2:**
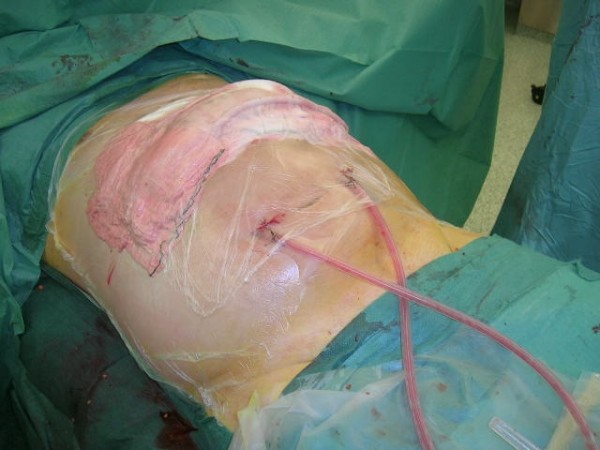
**Covering transverse laparostomy with "self-made" negative pressure dressing**.

The vacuum pack introduced in 1995 utilizes a polyethylene sheet tucked between the parietal peritoneum and the bowel, thus preventing the formation of adhesions between the abdominal wall and the bowel [10]. In 2001 the commercial vacuum-assisted wound management technique was introduced into everyday practise [11]. It helps the nursing care, but it has not been shown to be superior to various "self-made" applications used world-wide [12] (Fig. 2). Nevertheless, even in the management of the most severe complication of the open abdomen, the exposed enteric fistula, vacuum-assisted wound management is able to control the fistula secretion allowing the wound around it to heal [13,14].

**Figure 3:**
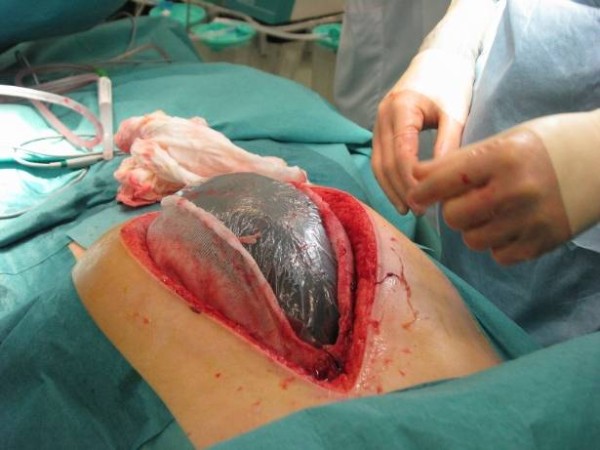
**Vacuum-dressing with temporary mesh being changed**.

Recently, a technique combining vacuum-dressing and a temporary mesh has been described [15]. It allows the gradual approximation of the fascial edges at every dressing change and eventual removal of the mesh and primary fascial closure (Fig. 3).

## Primary fascial closure

Once the ACS has been treated, the most important aim is to achieve primary fascial closure as soon as possible without causing recurrent ACS or other complications associated with premature closure [16]. If the infection source has been controlled and even if a relaparotomy might be needed in the near future, every effort should be made to achieve primary fascial closure during the initial hospitalization period and avoid the significant morbidity associated with leaving the abdomen open for delayed reconstruction. Gradual fascial closure, often mesh-assisted, seems to be the best currently available technique, but other possibilities, such as the components separation technique at an early stage [17], or fascial closure with a mesh prosthesis can be considered under favourable conditions (no infection, enough skin to cover the prosthesis). However, if primary fascial closure is not possible, an early decision to resort to the planned hernia strategy is a good option.

## Planned hernia

A planned hernia approach aims at skin coverage with subsequent delayed abdominal wall reconstruction. The skin closure is most often achieved with autologous split-thickness skin grafting over the exposed bowel. Instead of allowing the bowel surface to granulate before skin grafting, early application of the skin graft over the bowel seems to enhance the tuning down of the inflammatory process sustained by the large raw surface, and to make the subsequent reconstruction process easier.

The conditions favouring a planned hernia strategy include the inability to reapproximate the retracted abdominal wall edges, sizeable tissue loss, risk of tertiary abdominal compartment syndrome, inadequate infection source control, anterior enteric fistula and poor nutritional status of the patient. One of the factors that must also be taken into account is the type of the abdominal wall defect, especially if it is the result of decompressive laparostomy. A midline laparostomy covered with skin graft is relatively easily tolerated, whereas one from transverse laparostomy might cause serious disability due to the loss of abdominal and back extensor muscle functions, if fascial closure can not be achieved [6]. This might require complex reconstruction procedures to restore not only continuity but also the functional integrity of the abdomen [5]. If abdominal decompression has been achieved with the SLAF technique, the subsequent hernia can be corrected in a standard fashion utilizing prosthetic mesh or the components separation technique.

The maturation of the skin graft requires about 9–12 months, after which the grafted skin can be easily removed from the bowel surface without additional iatrogenic lesions. Large abdominal wall defects can be reconstructed with pedicular or microvascular flaps. The most commonly used is the tensor fascia lata (TFL)-flap [18]. With smaller defects, the components separation technique or a mesh repair can be also used for late repair provided that there is enough original skin for skin closure.

## Conclusion

IAH is a recently popularized clinical entity that is easily recognized, when suspected, using intravesicular measurement of IAP. The clinically significant manifestation, ACS, requires prompt attempts to reduce IAP starting with nonoperative measures. If these are insufficient, surgical decompression is warranted. The choice of the decompressive technique must be individualized. If a full-thickness laparostomy is performed, primary fascial closure should be attempted during the initial hospitalization period. If not possible, early resort to the planned hernia strategy and subsequent abdominal wall reconstruction are the best options provided that sufficient expertise is available.

## Competing interests

The author declares that they have no competing interests.

## Consent section

Written informed consents were obtained from the patients for publication of this review and accompanying images. Copies of the written consents are available for review by the Editor-in-Chief of this journal.
